# Supplementary material for: Distinct magneto-optical response of Frenkel and Wannier excitons in CrSBr
Source: Nat Commun. 2026 Jan 17;17:1777. doi: 10.1038/s41467-026-68482-5 (PMC12916971; doi:10.1038/s41467-026-68482-5)
Supplement: Supplementary file 1 — Supplementary Information [file 41467_2026_68482_MOESM1_ESM.pdf]

Distinct Magneto-Optical Response of Frenkel and Wannier Excitons in CrSBr - Supplementary Information

Maciej Śmiertka,[1] Michał Rygała,[2] Katarzyna Posmyk,[1,2] Paulina Peksa,[1,2] Mateusz Dyksik,[1] Dimitar Pashov,[3] Kseniia Mosina,[4] Zdenek Sofer,[4] Mark van Schilfgaarde,[5] Florian Dirnberger,[6,7,8] Michał Baranowski,[1,*] Swagata Acharya,[9,†] and Paulina Plochocka[1,2,‡]

[1] *Department of Experimental Physics, Faculty of Fundamental Problems of Technology, Wroclaw University of Science and Technology, 50-370 Wroclaw, Poland*
[2] *Laboratoire National des Champs Magnétiques Intenses, EMFL, CNRS UPR 3228, Université Grenoble Alpes, Université Toulouse, Université Toulouse 3, INSA-T, Grenoble and Toulouse, France*
[3] *King's College London, Theory and Simulation of Condensed Matter, The Strand, WC2R 2LS London, UK*
[4] *Department of Inorganic Chemistry, University of Chemistry and Technology Prague, Technicka 5, Prague 6, 16628 Czech Republic*
[5] *National Renewable Energy Laboratory, Golden, 80401, CO, USA*
[6] *Physics Department, TUM School of Natural Sciences, Technical University of Munich, Munich, Germany*
[7] *Zentrum für QuantumEngineering (ZQE), Technical University of Munich, Garching, Germany*
[8] *Munich Center for Quantum Science and Technology (MCQST), Technical University of Munich, Garching, Germany.*
[9] *National Renewable Energy Laboratory, Golden, 80401, CO, USA*
(Dated: December 17, 2025)

SUPPLEMENTAL MATERIALS

In systems with strongly localized electron and hole wavefunctions, atom-local excitons can be realized. Atomic multiplet transitions can have both excitonic and bi-excitonic characters [1]. These excitons are often refereed as the Frenkel excitons. The ground state excitons realized in the CrX_3 systems have strong Frenkel character and they are essentially atomic multiplets of Cr^{3+} ion [2]. For example, the 1.3 eV transition in $CrBr_3$ can be called an ideal Frenkel exciton that emerges from several valence and conduction bands and electrons and holes from all k points in the Brillouin zone take part in its formation (see Fig. S1). It is in that sense that in chemistry when we discuss atomic multiplets, band gap is often not a relevant parameter of interest, since for an atomic transition involving orbital characters that can spread over several bands, band gap does not remain a well defined quantity any more. Hence, orbitals that are involved in an atomic excitonic transition becomes the key ingredient for discussion. This fundamental assumption that considers that it is the atomic orbitals that determine the mutiplets lie at the heart of ligand-field theory and molecular-orbital description of multiplet transitions in highly localized systems. For example, in CrX_3 the ground state excitons can be described as multiplet transitions that involve t_{2g} holes and e_g electrons. When this is visualized in the band basis it looks like the Fig. S1 and has also been discussed in detail in our previous work on CrX_3 [2]. However for the higher energy excitonic transitions, the Frenkel character reduces and delocalized (in real space) Wannier character enhances [3] and excitons become more localized in the band basis. When the band edges take part in the exciton formation, the band gap becomes a more valid parameter for their description. It is in that sense that in the non-magnetic semiconductors and TMDs, excitonic description strictly involves a discussion of band gap and the binding energies for excitons with respect to the band gap. However, a $q = 0$ exciton does not necessarily involve the band edges at the high symmetry points and can be formed from different parts of the Brillouin zone and from different combinations of valence and conduction states. Our analysis suggests that the 1.3 eV exciton and its substructures have strong Frenkel character with strong analogy in a ligand-field theoretical framework that should describe those transitions. While for the 1.8 eV transition, the extended states are delocalized enough that they should be described within the Wannier-Mott framework that contains electron and hole excitations from the band-edge. Before we analyze the excitonic substructures we study in detail the orbital characters of the valence and conduction states. We see that the conduction band edge (see Fig. S2 and Fig. S3) is made of e_g states with the bottom most conduction state containing mostly $d_{x^2-y^2}$ character and the second (from bottom) conduction state

* michal.baranowski@pwr.edu.pl
† Swagata.Acharya@nrel.gov
‡ paulina.plochocka@lncmi.cnrs.fr

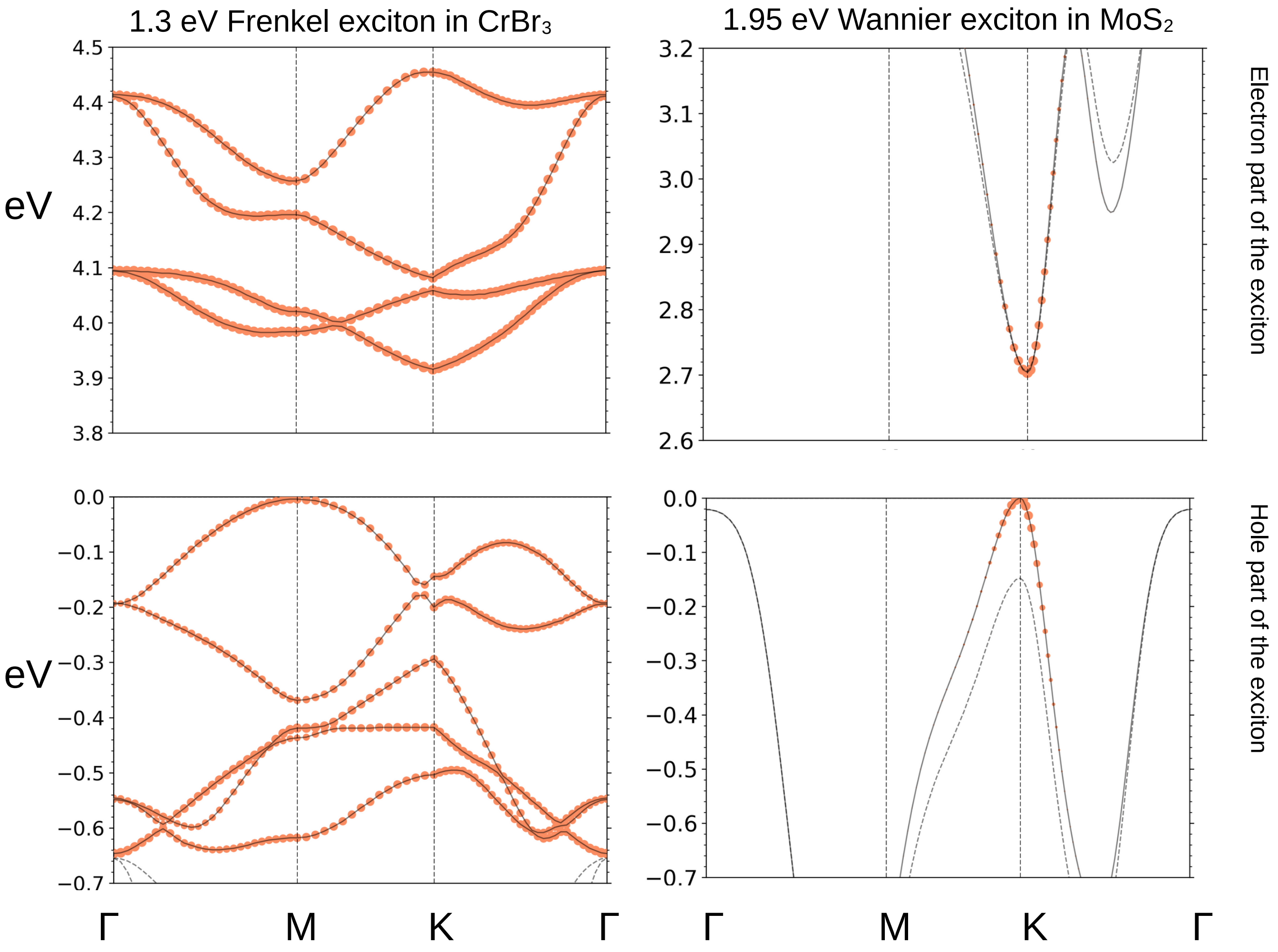

FIG. S1. **Frenkel and Wannier excitons in 2D magnets and TMD:** The excitonic wavefunction is projected on the bands. The hole part (valence) and electron part (conduction) of the wavefunctions are shown in violet while the bands in black don't contribute to the exciton formation. In $CrBr_3$ the ground state exciton is at 1.3 eV and a host of valence and conduction bands from the entire Brillouin zone take part in the exciton formation in strong contrast to the the 1.95 eV Wannier-Mott exciton in MoS_2 where only the band edges from the K point contribute to the exciton wavefunction.

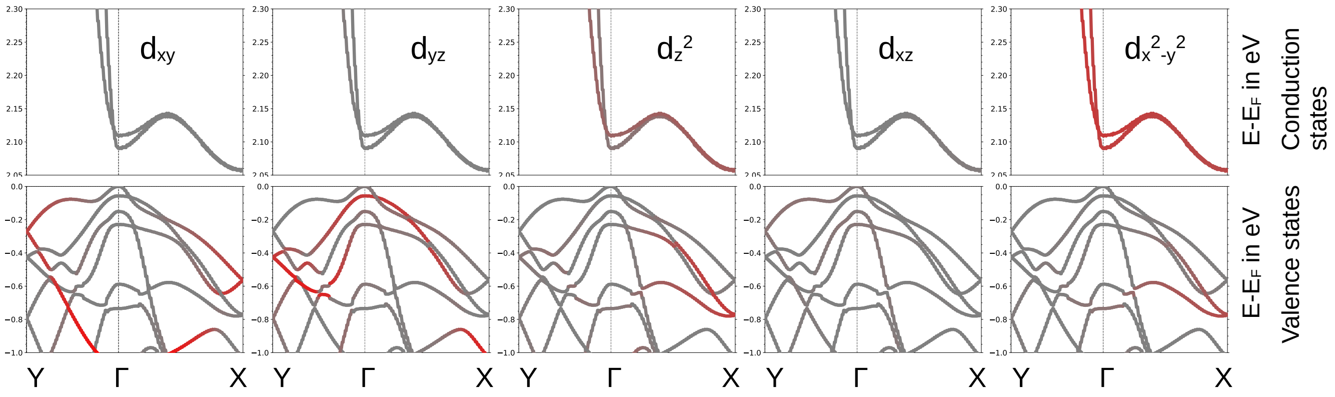

FIG. S2. **Orbital decomposition of the valence and conduction bands:** We observe that the valence states are made out of the t_{2g} orbitals, d_{xy}, d_{yz} with small contribution from d_{xz}. The conduction edge is made out of the e_g orbitals.

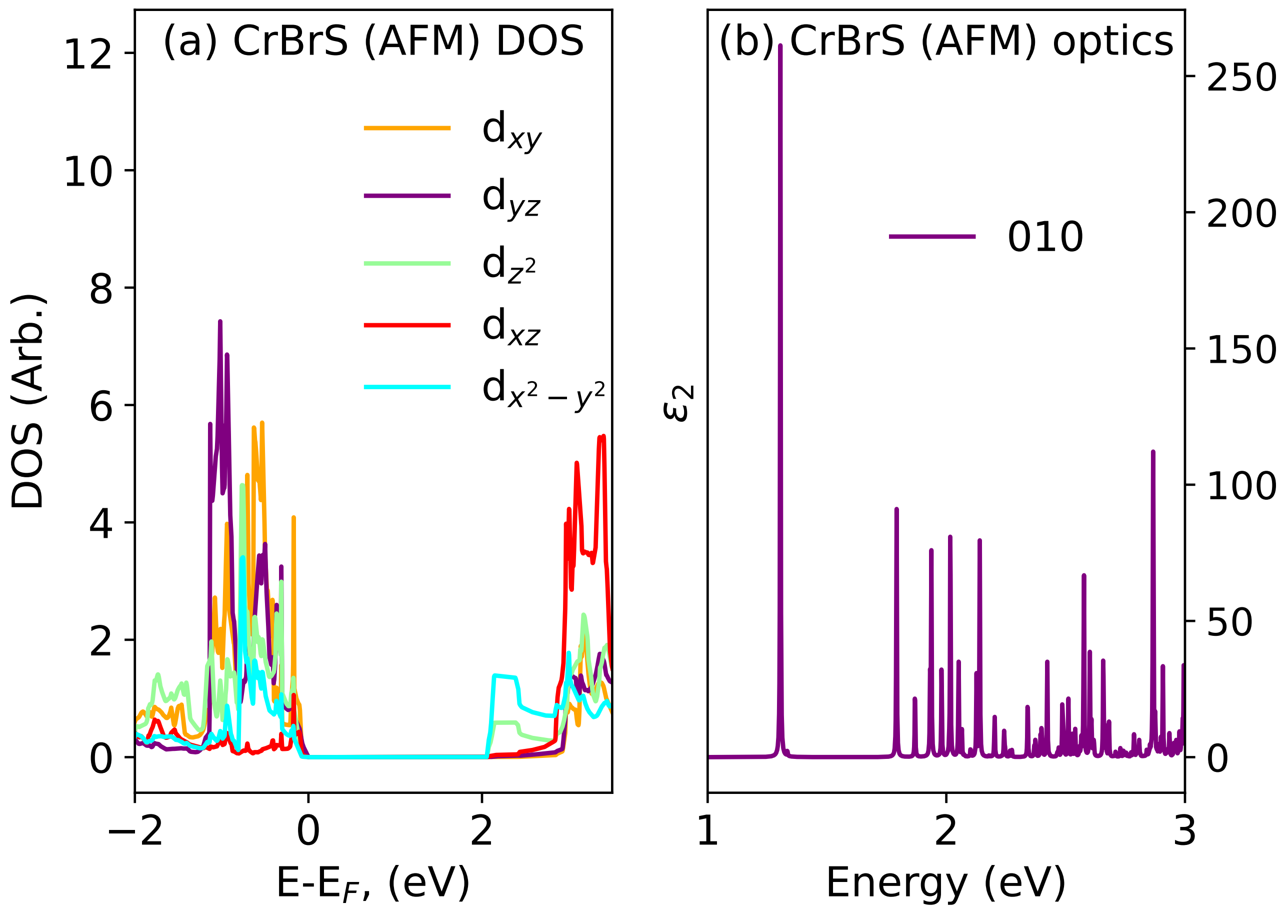

FIG. S3. **Orbitally projected density of states and the macroscopic dielectric response:** CrBrS valence states are made out of the t_{2g} orbitals and the conduction states are made out of the e_g orbitals. The strong $a-b$ structural anisotropy makes the d_{xz} state drop out of the top valence band states and simultaneously a large optical anisotropy is observed. The low energy excitonic manifold is dominated by response along 010 for the same reason. We plot the excitonic spectrum without including any optical broadening in the calculations so that all transitions can be identified clearly.

containing the d_{z^2} orbital character. The valence on the other hand contains mostly t_{2g}-d_{xy} and d_{yz} character. In a high symmetry crystal field, for example that of CrX_3, d_{yz} and d_{xz} should have similar contributions to the valence states, however, the large $a-b$ anisotropy of CrBrS implies that the d_{xz} contribution drops out from the top-most valence states and they become more d_{yz} like. This is in complete consistency with the observation that the optical response of the system at low energies are mostly along the b-xis and not along the a-axis (see Fig. S3). Further, the overall orbital symmetries of the valence and conduction states, with t_{2g} and e_g states of same spin is exactly what is expected for Cr^{3+} configuration.

Armed with this knowledge we analyze the excitons in the band and orbital basis next. We observe that X_A has two substructures. For both the transitions the hole is primarily contained in the Cr-d_{yz} orbital while it is the weakly split two conduction states of d_{z^2} and $d_{x^2-y^2}$ character respectively that lead to two excitonic transitions (see Fig. S4). In strong contrast, the substructures around the X_B transition mostly emerges from the valence and conduction edges (see Fig. S3) and have strong Wanner-Mott character. It is only natural that the holes are contained mostly in the d_{yz} orbitals the electrons come from the d_{z^2} and $d_{x^2-y^2}$ states as they are the dominant orbital characters of the valence and conduction edges (see Fig. S2 and Fig. S3). Having said that, it should be noted that these X_B transitions only have intersite character and should not be confused with large onsite *dd* character of the X_A transitions. Further, excitonic spectral weights can be observed at the valence band edge at the Γ point for some of the X_B substructures which has S-p character. In short, the origin of X_B substructures are significantly different from those of the X_A.

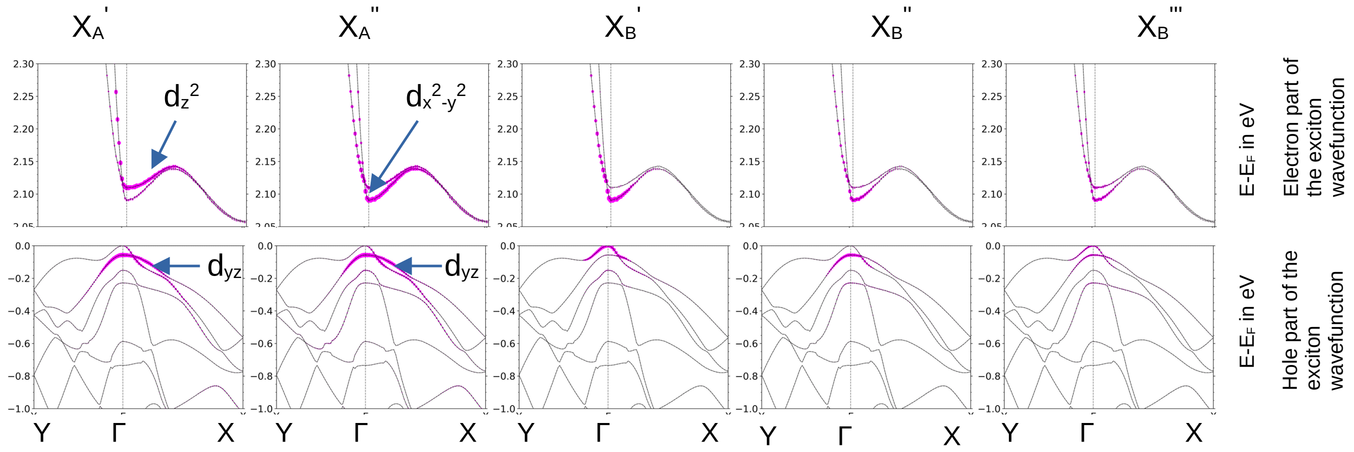

FIG. S4. **Excitonic substructure in CrBrS:** The spectral weight analysis for the excitonic substructure around the X_A and X_B transitions are shown. The projection is raised to its second power to show the concentration of the spectral weight on certain bands. The hole part (valence) and electron part (conduction) of the wavefunctions are shown in orange while the bands in black don't contribute to the exciton formation. There are two substructures to the X_A transition and both have large onsite *dd* character. One transition involves a hole d_{yz} state and an electron d_{z^2} state while the other transition involves a hole d_{yz} state and an electron $d_{x^2-y^2}$ state. The substructures of X_B are primarily band edge transitions involving t_{2g}-e_g inter-site transitions and dipolar S-p and Cr-d transitions with no onsite *dd* character.

Overall, the X_A and its substructures have strong Frenkel character the X_B and its substructures are more Wannier-Mott like. Having said that the X_A is not as Frenkel like as the $CrBr_3$ 1.3 eV Frenkel exciton and X_B is not as Wannier like as the MoS_2 1.95 eV exciton. Also, the substructures of these X_A and X_B transitions do not correspond to the Franck-Condon picture of molecular excitons which is often invoked in solid state systems with atomic multiplet transitions. The X_B and it's substructures don't have any analogy with the Rydberg series either.

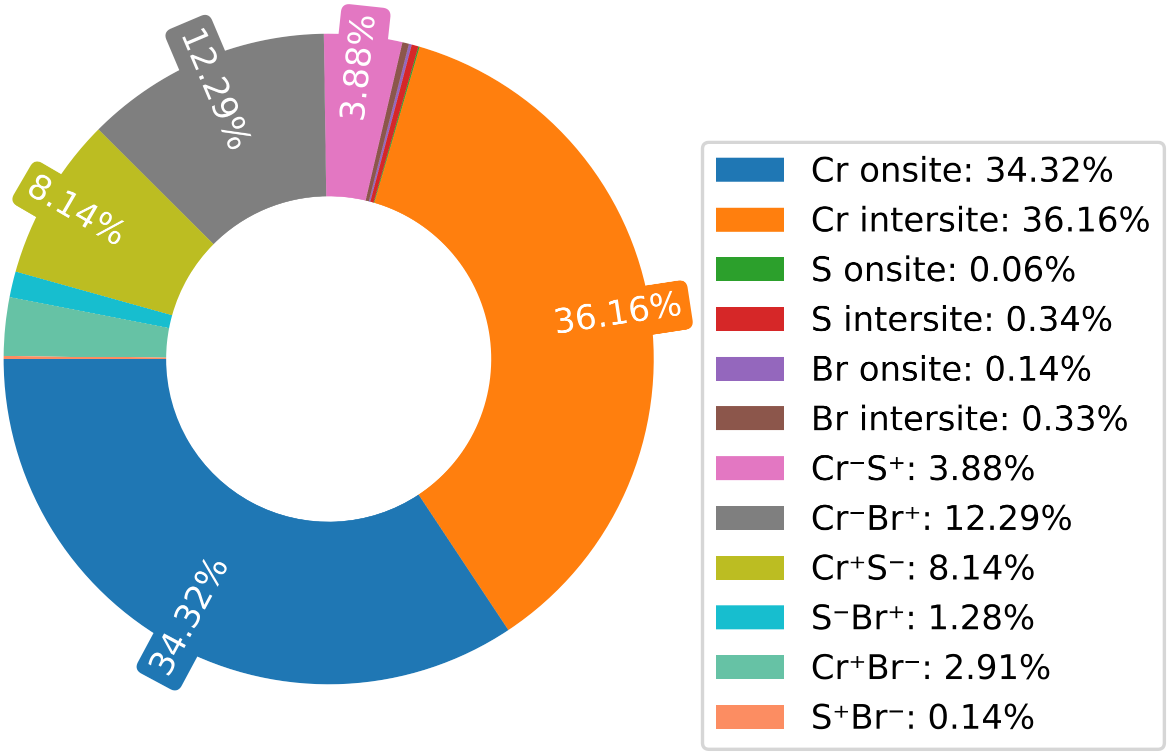

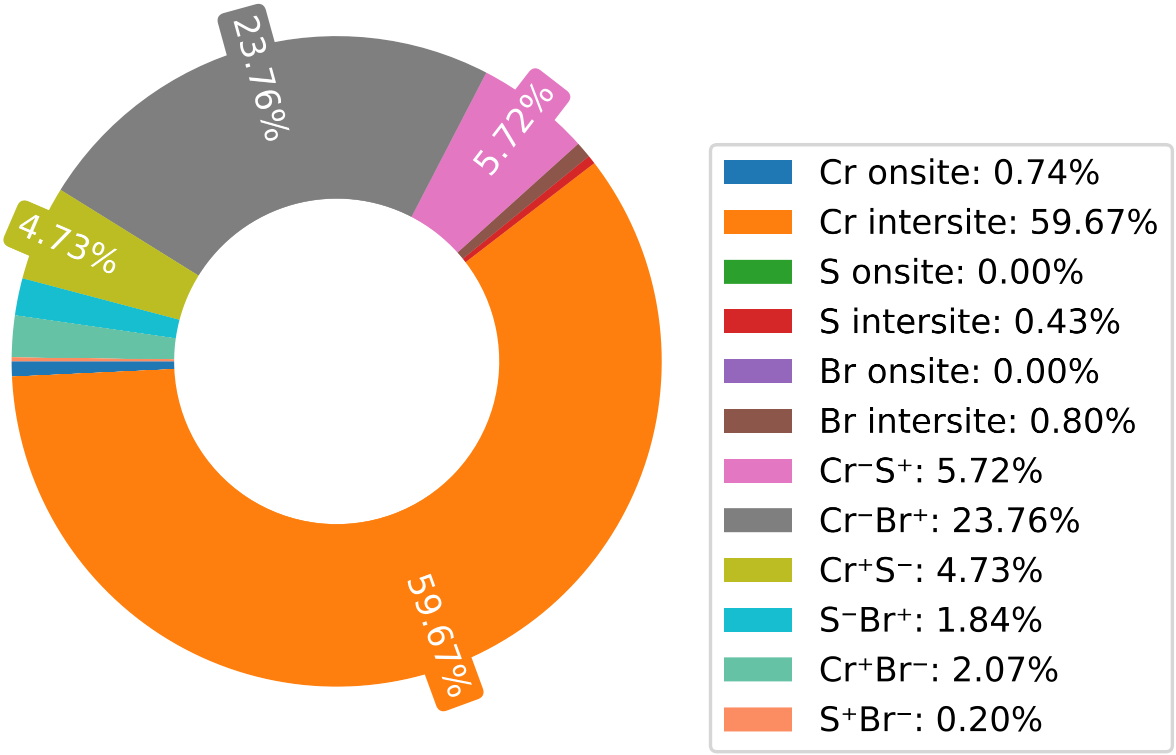

FIG. S5. The atomic and intra- and inter-site decompositions of the X_A and X_B transitions are shown in the FM phase.

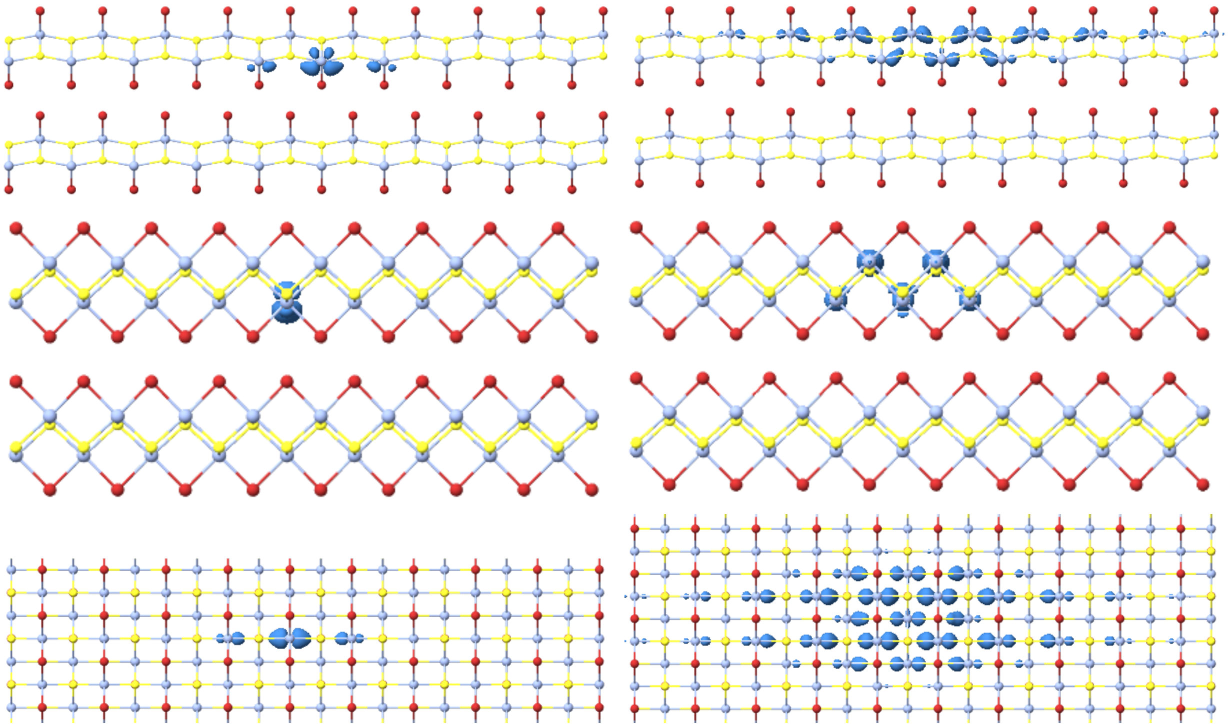

FIG. S6. Real space visualization of X_A and X_B in the AFM phase from different perspectives along x,y and z directions.

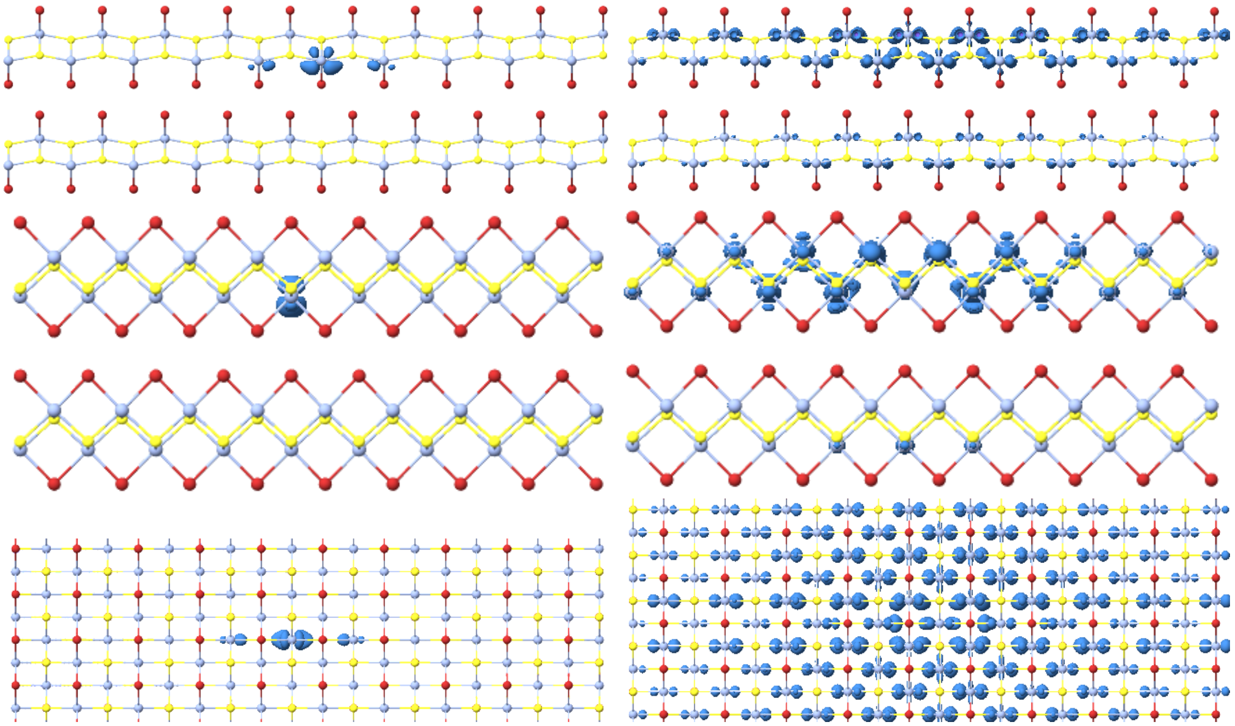

FIG. S7. Real space visualization of X_A and X_B in the FM phase from different perspectives along x,y and z directions.

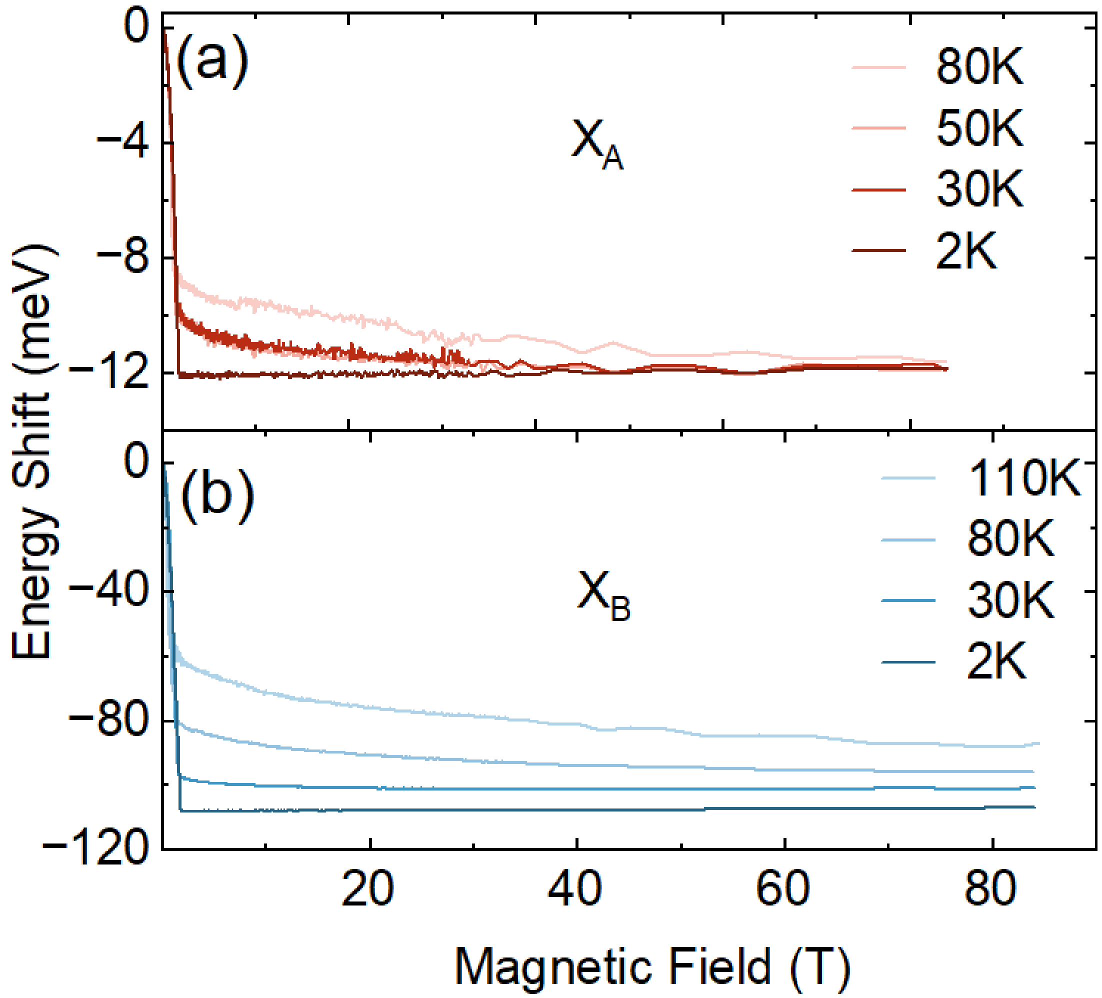

FIG. S8. Shifts of the excitonic transition as a function of the magnetic field, measured at different temperatures for (a) X_A and (b) X_B. For both transitions, saturation behaviour is observed in the high field limit; however, only for X_A the final redshift is temperature independent.

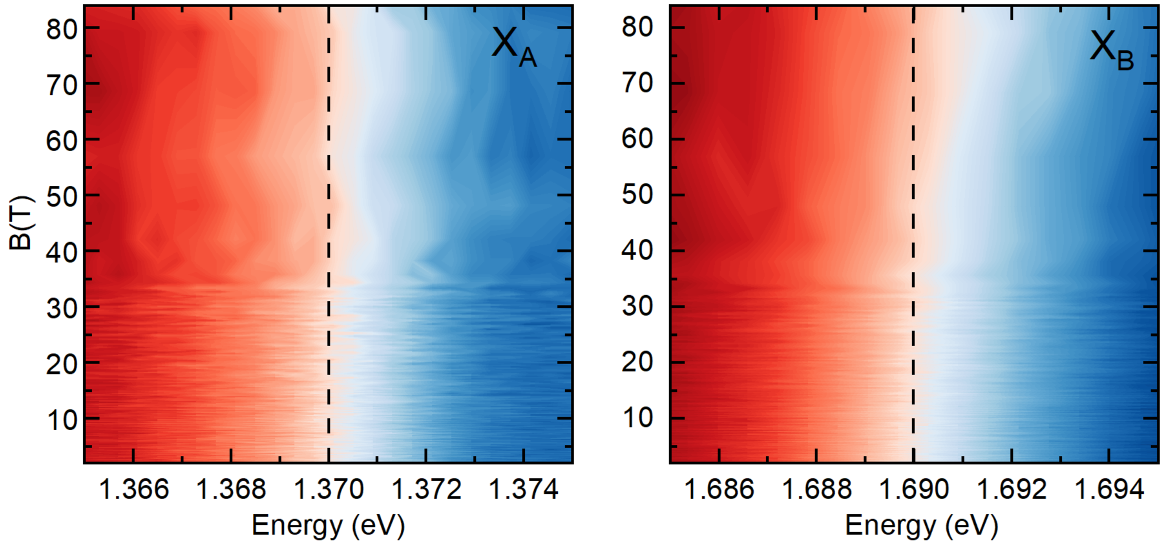

FIG. S9. Evolution of the reflectivity response, at 2K, as a function of the magnetic field in the energy range close to X_A and (b) X_B transition. For both transitions, a gentle blueshift is visible.

[1] S. Acharya, D. Pashov, C. Weber, M. van Schilfgaarde, A. I. Lichtenstein, and M. I. Katsnelson, A theory for colors of strongly correlated electronic systems, Nature Communications **14**, 5565 (2023).

[2] M. Grzeszczyk, S. Acharya, D. Pashov, Z. Chen, K. Vaklinova, M. van Schilfgaarde, K. Watanabe, T. Taniguchi, K. S. Novoselov, M. I. Katsnelson, and M. Koperski, Strongly Correlated Exciton-Magnetization System for Optical Spin Pumping in CrBr3 and CrI3., Advanced Materials **35**, 2209513 (2023).

[3] S. Acharya, D. Pashov, A. N. Rudenko, M. Rösner, M. v. Schilfgaarde, and M. I. Katsnelson, Real-and momentum-space description of the excitons in bulk and monolayer chromium tri-halides, npj 2D Materials and Applications **6**, 1 (2022).
